# Morphological and molecular characterization of myxobolids (Cnidaria, Myxozoa) infecting cypriniforms (Actinopterygii, Teleostei) endemic to the Iberian Peninsula

**DOI:** 10.1051/parasite/2019049

**Published:** 2019-08-16

**Authors:** Sónia Rocha, Carlos Azevedo, Ângela Alves, Carlos Antunes, Graça Casal

**Affiliations:** 1 Institute of Biomedical Sciences Abel Salazar, University of Porto (ICBAS/UP) Rua Jorge Viterbo Ferreira No. 228 4050-313 Porto Portugal; 2 Interdisciplinary Centre of Marine and Environmental Research (CIIMAR), Terminal de Cruzeiros de Leixões Av. General Norton de Matos s/n 4450-208 Matosinhos Portugal; 3 Aquamuseu do Rio Minho Parque do Castelinho 4920-290 Vila Nova de Cerveira Portugal; 4 University Institute of Health Sciences & Institute of Research and Advanced Training in Health Sciences and Technologies, CESPU Rua Central da Gandra No. 1317 4585-116 Gandra Portugal

**Keywords:** Myxosporea, Myxobolidae, Cypriniformes, phylogeny, SSU rDNA gene

## Abstract

The Iberian Peninsula provides a unique freshwater ecosystem for native and endemic cypriniforms to thrive. Despite cypriniforms being hosts to multiple myxobolids worldwide, little research has been performed in this geographic location. In this study, the examination of three Iberian endemic cypriniforms showed that myxosporean richness in the Iberian Peninsula is underestimated, with three new and one known myxobolid species being reported based on morphological and molecular data (SSU). *Myxobolus arcasii* n. sp. is described from the kidney and gonads of the “bermejuela” *Achondrostoma arcasii*, *M. duriensis* n. sp. from the gills of the Northern straight-mouth nase *Pseudochondrostoma duriense*, and *Thelohanellus paludicus* n. sp. from the intestine of the Southern Iberian spined-loach *Cobitis paludica*. *Myxobolus pseudodispar* Gorbunova, 1936 is further reported from several organs of *P. duriense*, and from the spleen of *A. arcasii*. The occurrence of *M. pseudodispar* in endemic Iberian species reveals that host-shift followed its co-introduction with central European leuciscids into this geographic location. Several other myxobolids originally described from barbels in central Europe have also been reported from the Iberian endemic cypriniform *Luciobarbus bocagei*. Nonetheless, except for *M. musculi*, the identification of these myxobolids in *L. bocagei* is here shown to be dubious and require molecular confirmation. Phylogenetic analyses reveal *M. arcasii* n. sp. and *M. duriensis* n. sp. clustering within different lineages of leuciscid-infecting species, showing that myxobolids entered Leuciscidae as hosts multiple times during their evolution. Constituting the first myxobolid reported from the subfamily Cobitinae, *Thelohanellus paludicus* n. sp. stands alone in the tree topology.

## Abstract (fr)

La péninsule ibérique constitue un écosystème d’eau douce unique pour le développement des cypriniformes indigènes et endémiques. Bien que les cypriniformes soient les hôtes de plusieurs myxobolides dans le monde, peu de recherches ont été effectuées dans cette zone géographique. Dans cette étude, l’examen de trois cypriniformes endémiques ibériques a montré que la richesse en Myxozoa de la péninsule ibérique était sous-estimée, trois nouvelles espèces de myxobolides et une connue étant répertoriées sur la base de données morphologiques et moléculaires (SSU). *Myxobolus arcasii* n. sp. est décrit à partir des reins et des gonades d’*Achondrostoma arcasii*, *Myxobolus duriensis* n. sp. des branchies de *Pseudochondrostoma duriense* et *Thelohanellus paludicus* n. sp. de l’intestin de *Cobitis paludica*. *Myxobolus pseudodispar* Gorbunova, 1936 est également signalé chez plusieurs organes de *P. duriense* et de la rate d’*A. arcasii*. La présence de *M. pseudodispar* chez des espèces ibériques endémiques révèle qu’un changement d’hôte a suivi sa co-introduction avec des Leuscicidae d’Europe centrale dans cette zone géographique. Plusieurs autres myxobolides décrits à l’origine sur des barbillons d’Europe centrale ont également été signalés chez *Luciobarbus bocagei*, un cypriniforme ibérique endémique. Néanmoins, à l’exception de *M. musculi*, l’identification de ces myxobolides chez *L. bocagei* s’avère douteuse et nécessite une confirmation moléculaire. Les analyses phylogénétiques révèlent que *M. arcasii* n. sp. et *M. duriensis* n. sp. se groupent au sein de différentes lignées d’espèces infectant les Leuscicidae, ce qui montre que les myxobolides ont acquis des hôtes Leuciscidae à plusieurs reprises au cours de leur évolution. Constituant le premier myxobolide signalé dans la sous-famille Cobitinae, *Thelohanellus paludicus* n. sp. est isolé dans la topologie des arbres.

## Introduction

The family Myxobolidae Thélohan, 1892 is the largest among the Myxozoa, specifically due to the species-richness of the genera *Myxobolus* Bütschli, 1881, *Henneguya* Thélohan, 1892 and *Thelohanellus* Kudo, 1933. Species belonging to these three genera are most commonly reported from freshwater habitats, less frequently occurring in brackish and marine habitats. Of the about 1200 known species of myxobolids, a significant amount has been described from fish hosts of the order Cypriniformes [15–18, 79]. The latter constitutes the largest group of freshwater fishes worldwide, being distributed across Europe, Asia, Africa and North America [19, 66]. Despite having highly diversified lifestyles, cypriniforms are restricted to freshwater and, therefore, can naturally expand their distribution only through the direct connection of habitats [66, 72]. The historical shaping of continental lands and inland waters thus influenced the evolution and radiation of this fish group [38, 66], which accounts for numerous species that are endemic to specific geographic locations.

The Iberian Peninsula has one of the greatest European percentages of endemism, not only due to the isolation caused by the Pyrenees and the Straits of Gibraltar, but also due to its complex fluvial network, which comprises a high number of independent river basins, in which freshwater communities are strongly isolated, and structured according to the orographic and climatic specificities of the region [9, 11, 28]. The majority of the native freshwater fish species in this geographic region belong to the order Cypriniformes, more specifically to the families Cyprinidae Rafinesque, 1815, Cobitidae Swainson, 1838, and Nemacheilidae Regan, 1911; with few representatives of the orders Acipenseriformes, Anguilliformes, Atheriniformes, Clupeiformes, Cyprinidontiformes, Petromyzontiformes, and Salmoniformes. Endemic species are found among representatives of the orders Cypriniformes and Cyprinidontiformes [23, 28], and include the bermejuela *Achondrostoma arcasii* (Steindachner, 1866) (Cypriniformes, Leuciscidae), the Northern straight-mouth nase *Pseudochondrostoma duriense* (Coelho, 1985) (Cypriniformes, Leuciscidae), and the Southern Iberian spined-loach *Cobitis paludica* (de Buen, 1930) (Cypriniformes, Cobitidae, Cobitinae). These three species are currently classified as vulnerable according to IUCN criteria and face massive conservation threats, namely due to the loss of spawning habitats and feeding grounds caused by severe anthropogenic changes of freshwater ecosystems. These include not only pollution and intense alterations, but also the introduction and spread of non-native species [3, 11, 12].

The acquisition of knowledge pertaining to parasites of native and endemic fish species is of major importance for gaining awareness of ecological issues in freshwater ecosystems, as parasites are useful indicators of the health of wild fish populations, as well as of habitat quality (e.g., [27, 31, 44, 70, 75]). Nonetheless, and despite the recognized importance of myxozoans as fish pathogens worldwide [34, 41, 42, 78], few studies concern the myxozoan community infecting native and endemic freshwater fishes of the Iberian Peninsula.

In the present study, microscopic and molecular descriptions are provided for three new myxobolids infecting cypriniforms endemic to the Iberian Peninsula. The muscle dwelling *Myxobolus pseudodispar* is further reported from two of the endemic fish species analyzed, revealing that host-shift occurred following co-introduction of the parasite into the Iberian Peninsula. Overall, the myxobolid biodiversity currently known from endemic fishes in this geographic area is shown to be underestimated.

## Materials and methods

### Fish sampling, myxozoan survey, and morphological analysis

Between 2013 and 2017, trimestral samplings of fish were performed from fyke-nets located in the River Minho (41°56′ N, 08°45′ W), near the border village of “Vila Nova de Cerveira”, Portugal. The River Minho marks the boundaries between northern Portugal and the Spanish autonomous community of Galicia. It originates in “Serra da Meira”, in the province of Lugo (Spain), and runs more than 300 km to drain into the Atlantic Ocean at the Portuguese northwest coast, near the village of “Caminha”. Fish samples included specimens of three cypriniform species endemic to the Iberian Peninsula: *Achondrostoma arcasii* (Steindachner, 1866) (*n* = 5; total length 13.2 ± 1.9 [9.9–14.4] cm; weight 33.2 ± 13.8 [10.0–47.0] g); *Pseudochondrostoma duriense* (Coelho, 1985) (*n* = 15; total length 22.5 ± 8.0 [9.3–33.0] cm; weight 137.8 ± 103.0 [6.0–349] g); and *Cobitis paludica* (de Buen, 1930) (*n* = 27; total length 9.2 ± 1.0 [7.0–11.2] cm; weight 5.2 ± 2.5 [2.0–12.0] g). Specimens were transported live to the laboratory and, prior to dissection, anesthetized with ethylene glycol monophenyl ether (Merck, Germany) at 1 mL/L. Several organs and tissues were macro- and microscopically examined for the presence of myxozoan parasites. Cysts and myxospores were photographed using an Olympus BX41 light microscope (Olympus, Japan). Morphometry was determined from fresh material, according to the guidelines provided by Lom and Arthur [40]. All measurements include the mean (*M*) value ± standard deviation (*SD*), range of variation, and number of myxospores measured (range, *n*). Prevalence of infection includes the mean value and confidence interval [CI] values.

### DNA extraction, amplification, and sequencing

Cysts and fragments of tissues containing myxospores were preserved in absolute ethanol at 4 °C. Genomic DNA extraction was performed using a GenElute™ Mammalian Genomic DNA Miniprep Kit (Sigma-Aldrich, St Louis, USA), following the manufacturer’s instructions.

The SSU rDNA gene was amplified using both universal and myxosporean-specific primers: the 5′-end by pairing the primer 18E (5′–CTG GTT GAT CCT GCC AGT–3′) [29] with the primers MyxospecR (5′–CAA CAA GTT GAT AGG GCA GAA–3′) [22], ACT3r (5′–ATT GTT CGT TCC ATG–3′) [62] and MYX4R (5′–CTG ACA GAT CAC TCC ACG AAC–3′) [26]; and the 3′-end by pairing the primers MyxospecF (5′–TTC TGC CCT ATC AAC TTG TTG–3′) [22], ACT3f (5′–CAT GGA ACG AAC AAT–3′) [26] and MYX4F (5′–GTT CGT GGA GTG ATC TGT CAG–3′) [63] with the primer 18R (5′–CTA CGG AAA CCT TGT TAC G–3′) [77]. PCRs were performed in 50 μL reactions using 10 pMol of each primer, 10 nMol of each dNTP, 2.0 mM MgCl_2_, 5 μL 10 × *Taq* polymerase buffer, 2.5 units *Taq* DNA polymerase (NZYTech, Lisbon, Portugal), and approximately 50–100 ng of genomic DNA. The reactions were run on a Hybaid PxE Thermocycler (Thermo Electron Corporation, Milford, MA, USA), with initial denaturation at 95 °C for 3 min, followed by 35 cycles of 94 °C for 45 s, 53 °C for 45 s, and 72 °C for 90 s. The final elongation step was performed at 72 °C for 7 min. Five-μL aliquots of the PCR products were electrophoresed through a 1% agarose 1 × tris-acetate-EDTA buffer (TAE) gel stained with ethidium bromide. PCR products were purified using Puramag™ magnetic beads coated with carboxylic acid groups (MCLAB, San Francisco, CA, USA).

The PCR products from different regions of the SSU rDNA gene were sequenced directly. The sequencing reactions were performed using a BigDye Terminator v3.1 Cycle Sequencing Kit from Applied Biosystems (Thermo Fisher Scientific, Waltham, MA, USA), and were run on an ABI3700 DNA analyzer from Applied Biosystems (Thermo Fisher Scientific, Waltham, MA, USA).

### Sequence assembly, distance estimation, and phylogenetic analysis

The partial sequences obtained for the different case isolates were aligned and assembled in MEGA 6.06 [73]. In order to calculate distance estimations, newly generated sequences were submitted to BLAST search (NCBI) to retrieve the SSU rDNA sequences with the highest similarity score. Other sequences belonging to congeners reported to infect the same host, or closely related hosts, were also included in the analysis, except for those which were deemed invalid by Molnár [51], as are the cases of the sequences of *Myxobolus impressus* (AF507970) and *Myxobolus dogieli* (EU003977, EU003978). The selected sequences were then aligned using the software MAFFT version 7 available online, and distance estimation was performed in MEGA 6.06, with the *p*-distance model and all ambiguous positions removed for each sequence pair.

For phylogenetic analysis, the dataset was widened to encompass the SSU rDNA sequences of other cypriniform-infecting myxobolids. Sequences belonging to species reported from the Iberian Peninsula were included, i.e. *M. branchialis* (Markevitsch, 1932), *M. branchilateralis* Molnár et al., 2012, *M. cutanei* Alvarez-Pellitero and González-Lanza, 1985, *M. leuciscini* González-Lanza and Alvarez-Pellitero, 1985, *M. musculi* Keysselitz, 1908, *M. pfeifferi* Thélohan, 1895, *M. pseudodispar* Gorbunova, 1936 and *M. tauricus* Miroshnichenko, 1979. The final dataset comprised a total of 67 SSU rDNA sequences, plus *Myxidium lieberkuehni* (X76638) and *Zschokkella auratis* (KC849425) as the outgroup. Alignments were performed using MAFFT software, version 7 available online, and posteriorly manually edited in MEGA 6.06. Phylogenetic trees were calculated from the sequence alignments using maximum likelihood (ML), maximum parsimony (MP), and Bayesian inference (BI). The general time reversible (GTR) substitution model with estimates of invariant sites and gamma distributed among site rate variation (GTR + *I* + Γ) was used in both ML and BI analyses, in accordance with the Modeltest algorithms of the software. BI analyses were performed using MrBayes v3.2.6 [65], with posterior probability distributions generated using the Markov Chain Monte Carlo (MCMC) method, with four chains running simultaneously for one million generations, and every 100th tree sampled. MP trees were obtained using the Subtree-Pruning-Regrafting algorithm with a search level of one and random initial tree addition of 10 replicates. Both ML and MP analyses were conducted in MEGA 6.06, with bootstrap confidence values calculated from 500 replicates.

## Results

### Myxozoan survey and overall prevalence of infection

Collected and analyzed fish specimens did not present obvious external symptoms of infection or disease. Macro- and microscopic analysis of 13 different organs revealed the presence of myxospores, disseminated or contained within cysts, in the gills, muscle, spleen, gonads, kidney, stomach, and intestine of several specimens. All myxospores were morphologically identified as belonging to the family Myxobolidae (phylum Cnidaria Hatschek, 1888, subphylum Myxozoa).

Only one out of the five specimens of *Achondrostoma arcasii* analyzed was simultaneously infected with two morphotypes of the genus *Myxobolus*. One morphotype formed cysts that were present in both the gonads and kidneys, while the other appeared disseminated in the spleen. Individual prevalence of infection of both morphotypes was 20.0% (one infected in five specimens analyzed). Molecular analysis of the SSU rDNA gene confirmed the presence of two distinct *Myxobolus* spp. infecting *A. arcasii*, with the morphotype in the gonads and kidneys being described here as a new species, and the one occurring in the spleen being identified as *M. pseudodispar* Gorbunova, 1936.

In turn, 14 out of the 15 specimens of *Pseudochondrostoma duriense* analyzed were infected by at least one of two *Myxobolus* morphotypes: one formed cysts in the gills, while the other appeared disseminated in the muscle, spleen, liver, kidneys, stomach and intestine. Overall prevalence of infection of *Myxobolus* in *P. duriense* was 93.3%; 53.3% (8 infected in 15 specimens analyzed) for the morphotype infecting the gills, and 86.7% (13 infected in 15 specimens analyzed) for the morphotype appearing disseminated in several tissues (see Table 1). Molecular analysis of the SSU rDNA gene confirmed the presence of two *Myxobolus* spp. infecting *P. duriense*, with the morphotype occurring in the gills being described here as a new species, and the other identified as *M. pseudodispar*.

**Table 1:** Presence/absence of *Myxobolus* infection in the organs of *P. duriense* examined, as determined by light microscopic observations.

Specimen#	Eye	Brain	Gills	Skeletal muscle	Heart	Liver	Gall bladder	Spleen	Swim bladder	Kidneys	Urinary bladder	Stomach	Intestine
1	–	–	–	–	–	–	–	–	–	–	–	–	Mp
2	–	–	–	–	–	–	–	–	–	–	–	Mp	Mp
3	–	–	Md	–	–	–	–	–	–	–	–	–	–
4	–	–	–	–	–	–	–	–	–	–	–	Mp	Mp
5	–	–	Md	–	–	Mp	–	Mp	–	–	–	–	Mp
6	–	–	–	–	–	–	–	–	–	Mp	–	–	–
7	–	–	Md	–	–	–	–	–	–	Mp	–	–	–
8	–	–	Md	–	–	–	–	–	–	–	–	–	–
9	–	–	–	–	–	–	–	Mp	–	–	–	Mp	–
10	–	–	Md	–	–	–	–	Mp	–	–	–	–	–
11	–	–	–	–	–	–	–	–	–	–	–	–	–
12	–	–	–	–	–	–	–	–	–	Mp	–	–	–
13	–	–	Md	–	–	–	–	–	–	Mp	–	–	Mp
14	–	–	Md	Mp	–	–	–	–	–	Mp	–	Mp	–
15	–	–	Md	–	–	–	–	Mp	–	–	–	–	–
PI	–	–	53.3%	6.7%	–	6.7%	–	26.7%	–	33.3%	–	26.7%	33.3%

PI, overall prevalence of infection of *Myxobolus* per organ examined; Md, *M. duriensis* n. sp.; Mp, *M. pseudodispar*.

Concerning the myxozoan survey performed on *Cobitis paludica*, only one representative of the genus *Thelohanellus* was found infecting the intestine of a single specimen and is described herein as a new species.

### Taxonomic position

Phylum Cnidaria Hatschek, 1888

Sub-phylum Myxozoa Grassé, 1970

Class Myxosporea Bütschli, 1881

Order Bivalvulida Shulman, 1959

Family Myxobolidae Bütschli, 1882

### *Myxobolus arcasii* n. sp.

urn:lsid:zoobank.org:act:B7BEA53C-5FBE-4C01-91F0-AA676AF4F8CE

Type host: *Achondrostoma arcasii* (Steindachner, 1866) (Cypriniformes, Leuciscidae) (common names: “bermejuela”, “ruivaco”, “panjorca” or “bogardo”).

Type locality: The River Minho (41°56′ N, 08°45′ W), near the village of “Vila Nova de Cerveira”, Portugal.

Site of infection: The hematopoietic tissue of the kidneys, and undifferentiated tissue of the gonads.

Prevalence of infection: 20.0% (one infected in five specimens analyzed; 95% CI [3.6, 62.5]).

Pathogenicity: Fish did not present evident external signs of infection or disease.

Type specimens: One glass slide containing semi-thin sections of the hapantotype was deposited in the Type Slide Collection of the Laboratory of Animal Pathology at the Interdisciplinary Centre of Marine and Environmental Research, Porto, Portugal, reference CIIMAR 2019.41.

Molecular data: Partial SSU rDNA gene sequence with a total of 1970 bp and GenBank accession no. MK053784.

Etymology: The specific epithet “*arcasii*” derives from the specific epithet of the host species.

**Figure 1:**
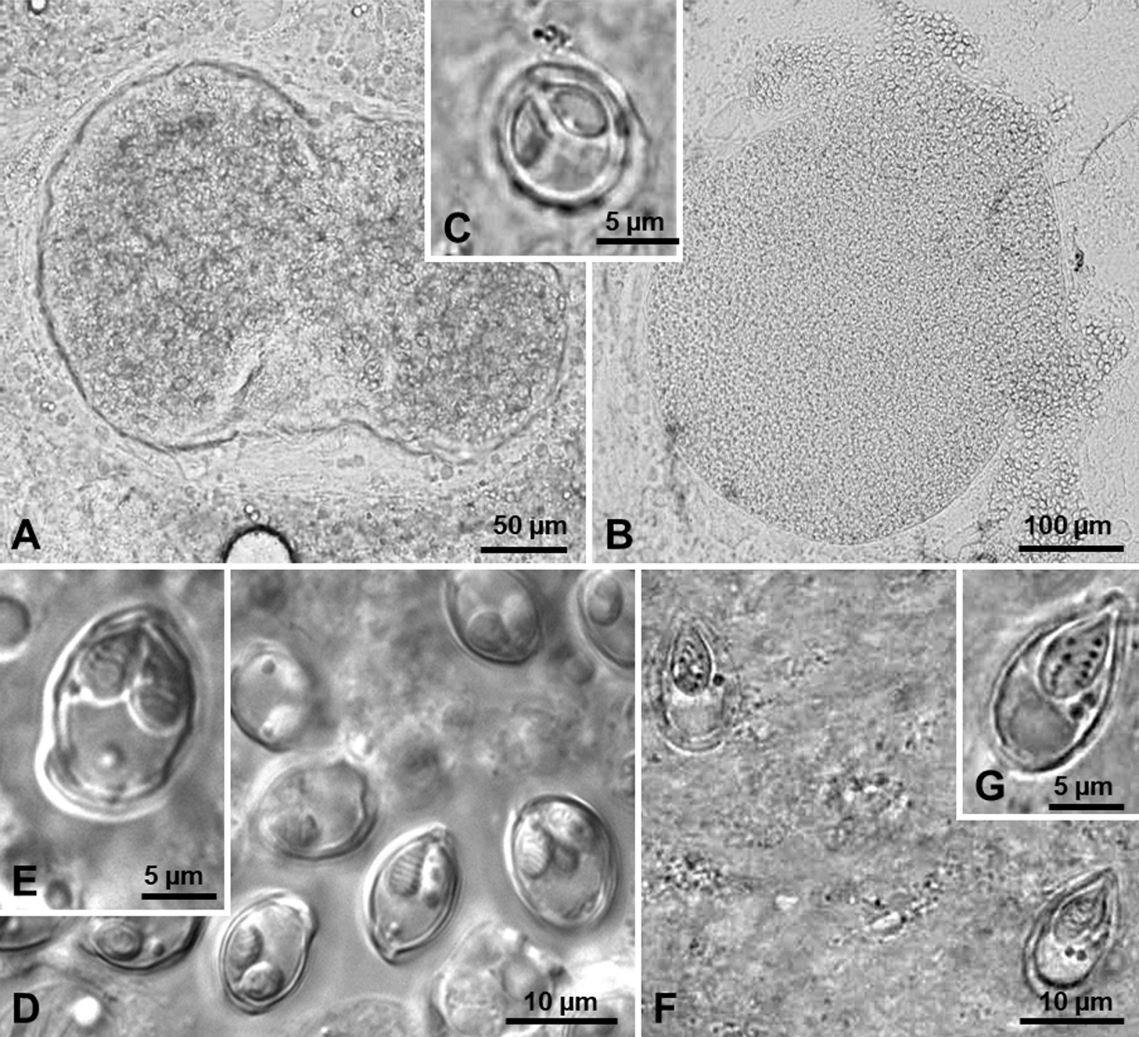
Light micrographs of wetmounts of the new myxobolid species found infecting Iberian endemic cypriniforms. (A–B) Cysts of *Myxobolus arcasii* n. sp. in the hematopoietic tissue of the kidneys, and in the undifferentiated tissue of the gonads of *Achondrostoma arcasii*, respectively. (C) Myxospore of *Myxobolus arcasii* n. sp. evidencing the two polar capsules in which the polar tubule coils. (D–E) Myxospores of *Myxobolus duriensis* n. sp. in the primary gill filaments of *Pseudochondrostoma duriense*, evidencing the polar tubule coiling within the polar capsules, and the iodinophilous vacuole in the sporoplasm. (F–G) Myxospores of *Thelohanellus paludicus* n. sp. in the intestinal epithelium of *Cobitis paludica*, showing the polar tubule coiling within the polar capsule, and the two conspicuous iodinophilous vacuoles in the sporoplasm.

**Figure 2:**
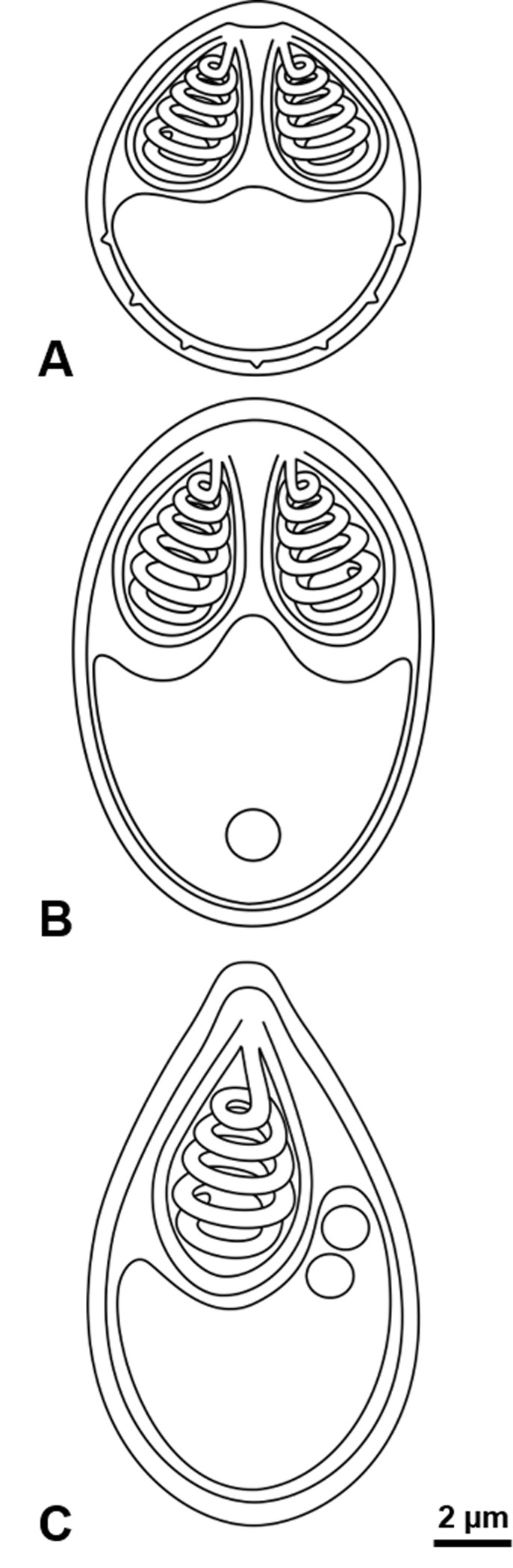
Schematic drawings of the new myxobolid species found infecting Iberian endemic cypriniforms; myxospores drawn in valvular view. (A) *Myxobolus arcasii* n. sp. (B) *Myxobolus duriensis* n. sp. (C) *Thelohanellus paludicus* n. sp.

Description (Figs. 1A–C and 2A).

Microscopic plasmodia of variable shapes and sizes in the hematopoietic tissue of the kidneys (Fig. 1A), and in the undifferentiated tissue of the gonads (Fig. 1B). Myxospores subspherical in valvular view and ellipsoidal in sutural view, 9.7 ± 0.5 (9.3–10.7) μm long (*n* = 15), 8.1 ± 0.2 (8.0–8.7) μm wide (*n* = 15), and 6.5 ± 0.2 (6.3–6.7) μm thick (*n* = 8). Valves smooth with 6–8 markings near the suture line. Two pyriform equal-sized polar capsules located side by side at the myxospore anterior pole, 3.9 ± 0.3 (3.3–4.3) μm long and 3.0 ± 0.2 (2.7–3.3) μm wide (*n* = 15). Polar tubule forming six (rarely seven) coils (Fig. 1C). Overall morphology is depicted in a schematic drawing representative of a myxospore in valvular view (Fig. 2A).

Remarks: Comparison of the parasite to other *Myxobolus* spp. reported from fishes of genera closely related to *Achondrostoma* Robalo et al., 2007 revealed highest morphometric similarity to *M. gallaicus* Iglesias et al., 2001, *M. leuciscini*, *M. bramae* Reuss, 1906 and *M. szentendrensis* Cech et al., 2015 (Table 2). The three latter species, however, can be readily distinguished from the parasite in study based on molecular data of the SSU rDNA gene. In turn, differentiation from *M. gallaicus* is established based on morphological traits; myxospores of *M. gallaicus* are slender with longer polar capsules and a higher number of polar tubule coils. Further comparison to the remaining *Myxobolus* spp. previously reported from more distantly related leuciscid genera revealed some morphometric similarity of the parasite to *M. hyborhynchi* Fantham et al., 1939 from bluntnose minnow *Pimephales notatus* (Rafinesque, 1820) in Canada, as well as to *M. schuberti* Li and Desser, 1985 and *M. siddalli* Salim and Desser, 2000 from common shiner *Luxilus cornutus* (Mitchill, 1817) in Canada (see [17]). Differentiation from *M. siddalli* could be performed by molecular comparison of respective SSU rDNA sequences. Despite lacking molecular data for comparison, *M. hyborhynchi* differs from the parasite in study in having significantly thinner polar capsules, while *M. schuberti* myxospores and polar capsules are generally longer and display a lower number of polar filament coils. Overall, distance estimation retrieved values of similarity consistently lower than 95.0%, including to *M. leuciscini* (91.5%), *M. bramae* (88.9%), *M. szentendrensis* (80.8%), and *M. siddalli* (91.0%). Considering all the above, this parasite is suggested as a new species, herein named *Myxobolus arcasii* n. sp.

**Table 2:** Comparison of *M. arcasii* n. sp. and *M. duriensis* n. sp. to other *Myxobolus* spp. reported from fishes belonging to closely related genera.

*Myxobolus* spp.	Hosts and location	Site of infection	SL	SW	ST	PCL	PCW	PTc	Source
*M. arcasii* n. sp.	*Achondrostoma arcasii* in Portugal	Kidneys and gonads	9.7 ± 0.5 (9.3–10.7)	8.1 ± 0.2 (8.0–8.7)	6.5 ± 0.2 (6.3–6.7)	3.9 ± 0.3 (3.3–4.3)	3.0 ± 0.2 (2.7–3.3)	6–7	Present study
*M. duriensis* n. sp.	*Pseudochondrostoma duriense* in Portugal	Gills	13.5 ± 0.3 (13.0–14.0)	9.0 ± 0.5 (8.0–9.7)	7.6 ± 0.3 (7.3–8.0)	4.9 ± 0.3 (4.3–5.3)	3.4 ± 0.1 (3.3–3.7)	6	Present study
*M. albovae* Krasilnikova in Shulman, 1966	*Leuciscus baicalensis*, and *Chondrostoma nasus* in the USSR	Gills	10.5–13.0	8.0–9.5	6.0–6.5	4.8–5.5	2.7–3.3	–	[17, 69]
*M. arrabonensis* Cech et al., 2015	*Chondrostoma nasus* in Hungary	Gills	8.7 ± 0.6 (8.4–10.0)	7.8 ± 0.3 (7.6–8.0)	5.5 (5.4–5.6)	4.8 ± 0.4 (4.5–5.9)	2.9 ± 0.1 (2.7–3.1)	6	[8]
*M. bliccae* Donec and Tozyyakova, 1984	*Blicca bjoerkna* in Ukraine, and other Eurasian cypriniforms, including *Chondrostoma nasus* in the USSR	Gills	12.4 ± 0.7 (10.5–14.0)	10.0 ± 0.7 (9.0–12.0)	6.6 ± 0.2 (6.0–7.0)	6.2 ± 0.5 (5.5–7.0)	3.4 ± 0.3 (3.0–4.0)	4–5	[13, 52]
*M. bramae* Reuss, 1906	*Abramis brama* in Russia, and other Eurasian cypriniforms, including *Chondrostoma nasus* in the USSR	Gills	10.0–12.0	8.0–10.0	4.5–6.5	4.0–5.5	2.3–3.5	4–5	[17, 25, 59, 69]
*M. chondrostomi* Donec, 1962	*Chondrostoma nasus* in Ukraine	Muscle	13.5–17.0	10.0–11.7	–	L 7.0–9.0	L 4.0–4.5	–	[17]
						S 5.5–7.2	S 3.0–3.5		
*M. donecae* Kashkovski in Shulman, 1966	*Leuciscus leuciscus* and *L. idus*, as well as in *Chondrostoma nasus* and *Tinca tinca* in the USSR	Gills, ureter	10.0–13.0	7.4–8.7	–	L 7.0	L 3.0	–	[13, 17]
						S 3.7	S 2.5		
*M. gallaicus* Iglesias et al., 2001	*Pseudochondrostoma polylepis* in Spain	Gills	10.0 (8.5–11.0)	8.8 (8.2–9.5)	5.7 (5.0–6.0)	4.9 (4.5–5.5)	2.9 (2.7–3.0)	7–8	[32]
*M. impressus* Miroshnichenko, 1980	*Barbus barbus* and *Squalius cephalus* in Ukraine. Also, *Abramis brama* in Hungary, and *Pseudochondrostoma polylepis* in Spain	Fins, gills	10.5–13.7	9.2–11.0	6.0–7.5	5.5–6.8	2.8–4.0	–	[17, 32, 59]
*M. leuciscini* González-Lanza and Alvarez-Pellitero, 1985^a^	*Squalius cephalus* in Hungary and probably Spain, and possibly in *Achondrostoma arcasii* and *Pseudochondrostoma polylepis* in Spain	Gills	10.5 ± 0.2 (10.0–11.5)	9.0 ± 0.2 (8.5–9.5)	6.3 ± 0.2 (6.0–6.5)	5.3 ± 0.2 (4.0–6.0)	2.9 ± 0.1 (2.5–3.2)	6–8	[25, 32, 58]
*M. lobatus* (Nemeczek, 1911) Landsberg and Lom, 1991	*Leuciscus leuciscus* and *L. aspius* in Germany, and also *Chondrostoma nasus* in the USSR	Gills	12.6	8.2	–	4.2	–	–	[17, 69]
*M. macrocapsularis* Reuss, 1906	*Blicca bjoerkna* in France, and other Eurasian cypriniforms, including *Chondrostoma nasus* in the USSR	Gills	13.6 ± 0.9 (12.0–16.0)	9.7 ± 0.7 (8.0–11.0)	6.2 ± 0.2 (6.0–6.5)	7.3 ± 0.6 (6.5–9.0)	3.4 ± 0.3 (3.0–4.0)	7	[13, 41, 43, 52]
*M. muelleri* Bütschli, 1882^b^	*Squalius cephalus*, and possibly in other Eurasian cypriniforms, including *Achondrostoma arcasii* and *Pseudochondrostoma polylepis* in Spain	Gills	9.8 ± 0.2 (9.5–10.0)	7.5 ± 0.2 (7.5–8.0)	5.2 ± 0.2 (5.0–5.5)	4.6 ± 0.5 (4.0–5.0)	3.6 ± 0.5 (3.0–4.0)	5–6	[17, 25, 33, 36, 41, 57]
*M. paksensis* Cech et al., 2015	*Chondrostoma nasus* in Hungary	Swimbladder	14.8 ± 0.6 (14.4–15.2)	11.0 ± 0.7 (10.4–12.0)	8.7 (8.4–9.2)	7.0 ± 0.4 (6.8–7.6)	4.3 ± 0.2 (4.0–4.6)	6	[8]
*M. pseudodispar* Gorbunova, 1936	*Rutilus rutilus*, and several other Eurasian cypriniforms, including *Achondrostoma arcasii* and *Pseudochondrostoma polylepis* in Portugal and Spain	Muscle, gills, kidney, urinary ducts, liver, spleen	10–12	7.0–9.5	5.3–6.0	L 4.5–6.2	L 3.0–3.7	–	[10, 17, 25]
						S 3.9–5.0	S 2.7–3.0		
*M. szentendrensis* Cech et al., 2015	*Chondrostoma nasus* in Hungary	Gills	9.2 ± 0.3 (8.8–9.6)	7.9 ± 0.7 (7.6–8.0)	6.7 (6.4–7.1)	5.3 ± 0.3 (4.8–5.6)	3.0 ± 0.2 (2.8–3.2)	6	[8]

SL, myxospore length; SW, myxospore width; ST, myxospore thickness; PCL, polar capsule length; PCW, polar capsule width; PTc, number of polar tubule coils; S, smaller; L, larger.

Measurements from infections in the type host are given in μm, as *M* ± *SD* (range) (when available).

a Measurements from the original description in *Squalius cephalus*, since *M. leuciscini* was morphologically and molecularly re-described from this fish host in Hungary [58].

b Data from the morphological re-description and molecular identification of the parasite from its original site of infection and host species in Hungary [57].

### *Myxobolus duriensis* n. sp.

urn:lsid:zoobank.org:act:3BE0E35E-8AFD-483C-8825-3FACB06EC99F

Type host: The Northern straight-mouth nase *Pseudochondrostoma duriense* (Coelho, 1985) (Cypriniformes, Leuciscidae) (common name: “boga”).

Type locality: The River Minho (41°56′ N, 08°45′ W), near the village of “Vila Nova de Cerveira”, Portugal.

Site of infection: The primary gill filaments.

Prevalence of infection: 53.3% (8 infected in 15 specimens analyzed; 95% CI [33.0, 83.0]).

Pathogenicity: Fish did not present evident external signs of infection or disease.

Type specimens: One glass slide containing semi-thin sections of the hapantotype was deposited in the Type Slide Collection of the Laboratory of Animal Pathology at the Interdisciplinary Centre of Marine and Environmental Research, Porto, Portugal, reference CIIMAR 2019.42.

Molecular data: Partial SSU rDNA gene sequence with a total of 1983 bp, deposited in GenBank with the accession no. MK053783. The latter is representative of four identical sequences that were separately assembled from the partial results obtained from cysts in the gills of four infected specimens.

Etymology: The specific epithet “*duriensis*” derives from the specific epithet of the host species.

Description (Figs. 1D–E and 2B).

Microscopic cysts, spherical to subspherical, in the primary gill filaments. Myxospores subspherical in valvular view and ellipsoidal in sutural view, with smooth valves, 13.5 ± 0.3 (13.0–14.0) μm long (*n* = 20), 9.0 ± 0.5 (8.0–9.7) μm wide (*n* = 20), and 7.6 ± 0.3 (7.3–8.0) μm thick (*n* = 13). Two pyriform equal-sized polar capsules located side by side at the myxospore anterior pole, 4.9 ± 0.3 (4.3–5.3) μm long and 3.4 ± 0.1 (3.3–3.7) μm wide (*n* = 25). Polar tubule forming six coils. One, more rarely two, small iodinophilous vacuoles in the sporoplasm (Fig. 1D–E). Overall morphology is depicted in a schematic drawing representative of a myxospore in valvular view (Fig. 2B).

Remarks: Comparison of the parasite in study to all other *Myxobolus* spp. previously described from leuciscid hosts, and more specifically to those reported from fishes of genera closely related to *Pseudochondrostoma* Robalo et al., 2007 (Table 2), revealed significant differences be it either in the morphology of the myxospores or molecular data of the SSU rDNA gene. Closest morphometric resemblance was determined in relation to *M. chernovae* (Chernova, 1970) Landsberg and Lom, 1991, *M. compressus* Kudo, 1934, *M. fanthami* (Fantham et al., 1939) Landsberg and Lom, 1991, *M. orbitalis* (Fantham et al., 1939) Landsberg and Lom, 1991, *M. parallelipticoides* (Fantham et al., 1939) Landsberg and Lom, 1991 and *M. pfrille* (Fantham et al., 1939) Landsberg and Lom, 1991, all of which were described from fish hosts belonging to other leuciscid genera in distant geographic locations. *M. chernovae* was described from roach *Rutilus rutilus* (Linnaeus, 1758) in Russia; *M. compressus* from River shiner *Notropis blennius* (Girard, 1856) in the USA; *M. fanthami* and *M. orbitalis* from common shiner *Luxilus cornutus* in Canada; and *M. parallelipticoides* and *M. pfrille* from finescale dace *Chrosomus neogaeus* (Cope, 1867), also in Canada (see [17]). Despite sharing some morphometric similarity with the parasite in study, the myxospores of *M. chernovae* are larger and display shorter polar capsules. In turn, those of *M. compressus*, *M. fanthami* and *M. orbitalis* have significantly thinner polar capsules, with the two latter further being generally bigger. The myxospores of *M. parallelipticoides* and *M. pfrille* also display thinner polar capsules and have a significantly wider morphometric range than those in study. Thus, this parasite is suggested as a new species, herein named *M. duriensis* n. sp. Distance estimation revealed highest similarity to *M. szentendrensis* (96.2%), with all other SSU rDNA sequences included in the analysis retrieving similarity values lower than 95.0%. Thus, this parasite is suggested as a new species, herein named *M. duriensis* n. sp.

### *Myxobolus pseudodispar* Gorbunova, 1936

**Figure 3:**
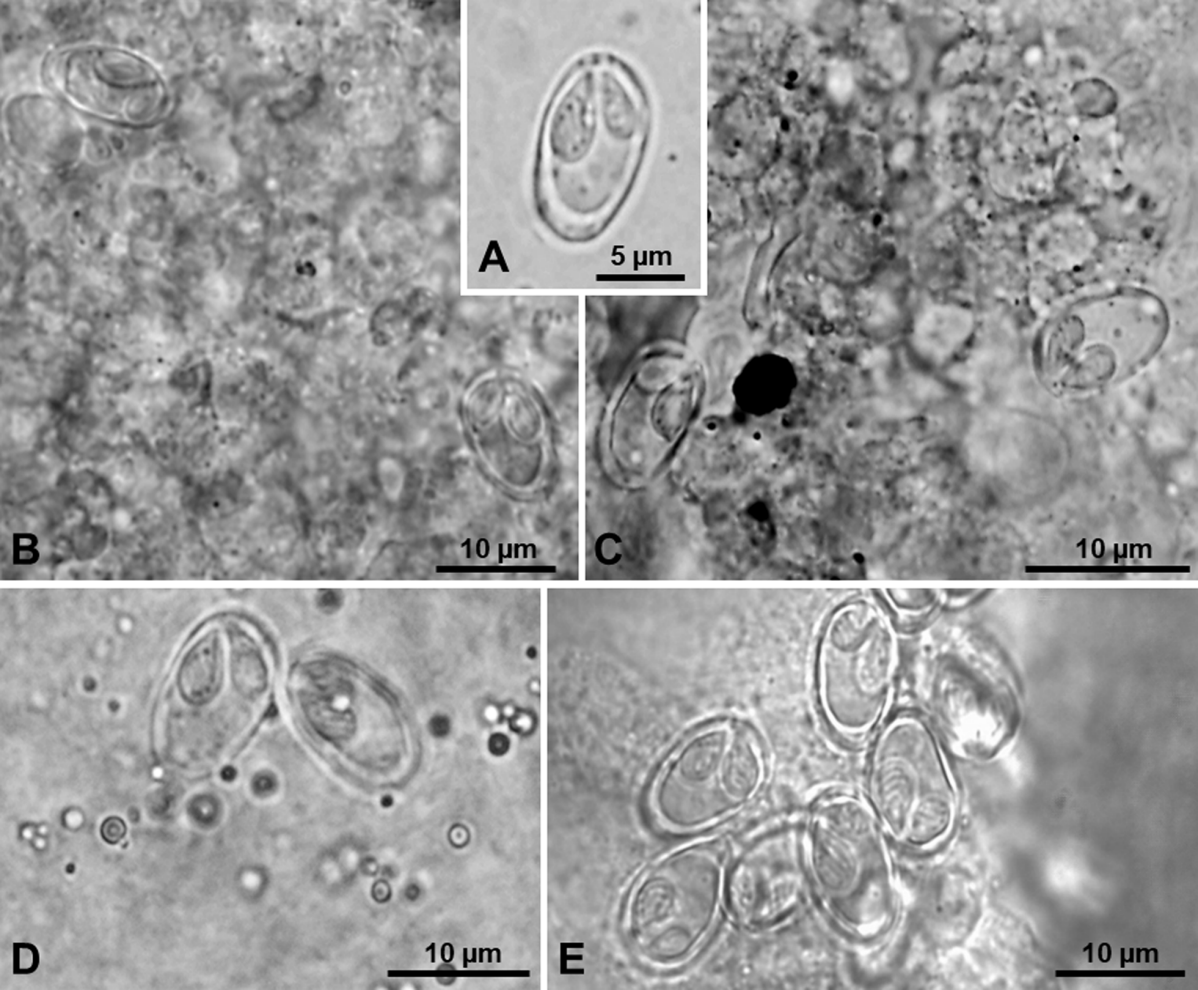
Light micrographs of myxospores of *Myxobolus pseudodispar* Gorbunova, 1936 infecting the connective tissue of several organs of Iberian endemic cypriniforms. (A) Myxospore evidencing the overall asymmetry of its morphological features. (B) Infection in the spleen of *Achondrostoma arcasii*. (C–E) Infection in the kidney, digestive tube, and muscle of *Pseudochondrostoma duriense*, respectively.

This cosmopolitan species was identified from infected tissue samples of both *P. duriense* and *A. arcasii*, based on the morphometric aspects of the myxospores (i.e. asymmetrical shape and polar capsules different in size), broad range of sites of infection, and molecular data of the SSU rDNA gene. Infection by this parasite was determined to occur in the muscle, spleen, liver, kidneys, stomach and intestine of 13 out of 15 specimens of *P. duriense* analyzed (86.7%; 95% CI [62.2, 96.3]), as well as in the spleen of a single individual out of the five specimens of *A. arcasii* analyzed (20.0%; 95% CI [3.6, 62.5]). Large, elongated plasmodia were observed in the muscle, while disseminated myxospores appeared located in the stomach and intestine, and in melanomacrophage centers of the spleen and renal parenchyma (Fig. 3). Myxospores were ellipsoidal with smooth valves, asymmetric, 10.9 ± 0.4 (10.0–11.3) μm long (*n* = 30) and 7.1 ± 0.3 (6.7–7.3) μm wide (*n* = 30). Two unequal polar capsules were pointed laterally to the left of the medial plane in valvular view. The polar tubule coiled obliquely in four (rarely five) turns (Fig. 3). In total, two partial SSU rDNA gene sequences belonging to this species were deposited in GenBank, under the accession no. MK053785 and MK024332. The first comprised a total of 1961 bp, being representative of the identical partial sequences obtained from the muscle, spleen, liver, kidneys, stomach, and intestine of different infected specimens of *P. duriense*; while the second comprised 1206 bp and was obtained from infected samples of spleen belonging to a single specimen of *A. arcasii*. These sequences shared 99.2% similarity amongst each other, accounting for nine nucleotide substitutions in a comparison established from 1206 bp. Distance estimation revealed similarity values ranging between 94.6–97.3% to Hungarian isolates of *M. pseudodispar* from the type host *Rutilus rutilus*, 94.2–98.7% to isolates from rudd *Scardinius erythrophthalmus* (Linnaeus, 1758), 96.2–98.2% to isolates from freshwater bream *Abramis brama* (Linnaeus, 1758), 95.2–97.9% to isolates from white bream *Blicca bjoerkna* (Linnaeus, 1758), and 96.3–96.5% to isolates from bleak *Alburnus alburnus* (Linnaeus, 1758).

### *Thelohanellus paludicus* n. sp.

urn:lsid:zoobank.org:act:5C7A0060-25FB-421E-A207-F973AED8652D

Type host: The Southern Iberian spined-loach *Cobitis paludica* (de Buen, 1930) (Cypriniformes, Cobitidae, Cobitinae) (common name: “verdemã do Norte”).

Type locality: The River Minho (41°56′ N, 08°45′ W), near the village of “Vila Nova de Cerveira”, Portugal.

Site of infection: The intestine.

Prevalence of infection: 3.7% (one infected in 27 specimens analyzed; 95% CI [0.6, 18.3]).

Pathogenicity: Fish did not present evident external signs of infection or disease.

Type specimens: One glass slide containing semi-thin sections of the hapantotype was deposited in the Type Slide Collection of the Laboratory of Animal Pathology at the Interdisciplinary Centre of Marine and Environmental Research, Porto, Portugal, reference CIIMAR 2019.43.

Molecular data: Partial SSU rDNA gene sequence with a total of 1602 bp and GenBank accession no. MK053786.

Etymology: The specific epithet “*paludicus*” derives from the specific epithet of the host species.

Description (Figs. 1F–G and 2C).

Plasmodia not observed. Individual myxospores disseminated in the intestinal epithelium. Myxospores ellipsoidal with a slightly more pointed anterior pole, with smooth valves, 14.4 ± 0.5 (14.0–15.3) μm long (*n* = 15) and 8.4 ± 0.3 (8.0–8.7) μm wide (*n* = 15). Single pyriform polar capsule, 6.7 ± 0.3 (6.0–7.0) μm long and 3.9 ± 0.1 (3.7–4.0) μm wide (*n* = 15), positioned slightly to the left of the medial plane in valvular view. Polar tubule forming 5–6 coils. Two to three conspicuous iodinophilous vacuoles in the sporoplasm (Fig. 1F–G). Overall morphology is depicted in a schematic drawing representative of a myxospore in valvular view (Fig. 2C).

Remarks: Currently, there are no reports of *Thelohanellus* spp. infecting fishes of the genus *Cobitis* Linnaeus, 1758, nor of any other fish genera in the Iberian Peninsula. Comparison to the three congeners reported from fish of the subfamily Cobitinae Swainson, 1838 worldwide, revealed *T. paludicus* n. sp. differing not only in host species and geographic location, but also in myxospore morphometry (Table 3). The myxospores and polar capsules of *T. paludicus* n. sp. are larger than those of *T. acuminatus* Akhmerov, 1955, a species that was originally described from the gills of common carp *Cyprinus carpio* Linnaeus, 1758 in Far-East Russia, and further reported from the pond loach *Misgurnus anguillicaudatus* (Cantor, 1842). In turn, despite *T. paludicus* n. sp. being overall morphometrically similar to *T. misgurni* (Kudo, 1919) Kudo, 1933, the latter was described from the gall bladder of *M. anguillicaudatus* in Tokyo and displays thinner myxospores. Finally, the measurements of *T. paludicus* n. sp. are within the wide morphometric range reported for *T. pyriformis* (Thélohan, 1892) Kudo, 1933; however, the high morphological variability found among the various reports of the latter species from different tissues and organs of several cyprinids across Europe (including the weatherfish *Misgurnus fossilis* [Linnaeus, 1758]), suggest that it is a possible species complex. Molecular comparison to these three species is not possible due to the lack of available information. Overall, distance estimation revealed *T. paludicus* n. sp. without significant percentage of similarity to any of the SSU rDNA sequences presently available for its congeners and other myxobolids in general.

**Table 3:** Comparison of *T. paludicus* n. sp. to other *Thelohanellus* spp. from cyprinid fish hosts of the subfamily Cobitinae.

*Thelohanellus* spp.	Hosts and location	Site of infection	SL	SW	ST	PCL	PCW	PTc	Source
*T. paludicus* n. sp.	*Cobitis paludica* in Portugal	Undifferentiated tissues of kidneys and gonads	14.4 ± 0.5 (14.0–15.3)	8.4 ± 0.3 (8.0–8.7)	–	6.7 ± 0.3 (6.0–7.0)	3.9 ± 0.1 (3.7–4.0)	5–6	Present study
*T. acuminatus Akhmerov, 1955*	*Cyprinus carpio* in Far-East Russia. Also *Misgurnus anguillicaudatus* in Far-East Russia	Gills	13.0–14.0	5.0	–	7.0–7.5	2.0–2.3	–	[79]
*T. misgurni* (Kudo, 1919) Kudo, 1933 [Syns *Myxobolus misgurni* Kudo, 1919; *M. fuhrmanni* Kudo, 1916]	*Misgurnus anguillicaudatus* in Japan	Gall bladder	14.0–15.5	6.0–7.3	2.0–3.0	6.3–7.5	3.7	4–5	[36, 37]
*T. pyriformis* (Thélohan, 1892) Kudo, 1933	*Tinca tinca*, and other Eurasian cypriniforms, including *Misgurnus fossilis*	Gills, spleen, liver, intestine, and kidneys	17.1 (14.9–22.0)	8.2 (6.1–9.7)	6.4 (6.0–6.9)	6.9 (5.1–10.6)	3.2 (2.3–4.5)	–	[36, 37, 79]

SL, myxospore length; SW, myxospore width; ST, myxospore thickness; PCL, polar capsule length; PCW, polar capsule width; PTc, number of polar tubule coils; S, smaller; L, larger.

Measurements are given in μm, as *M* ± *SD* (range) (when available).

### Phylogenetic analysis

**Figure 4:**
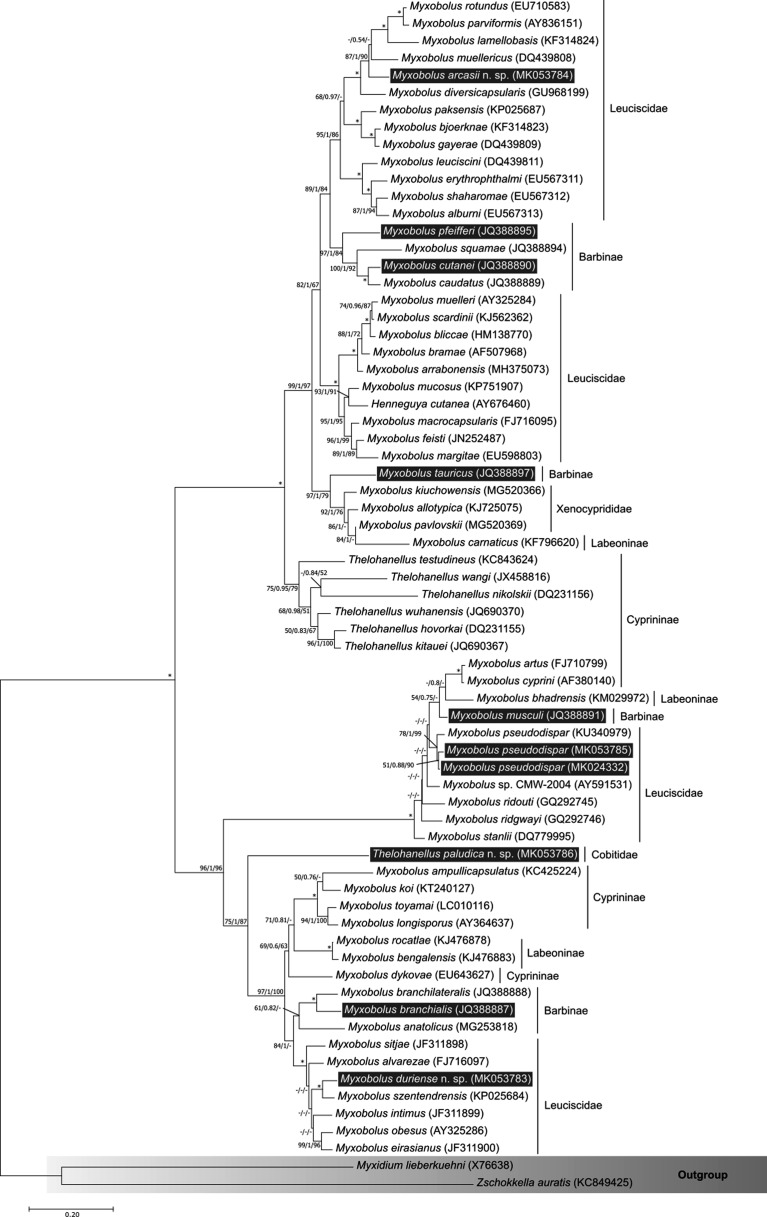
Tree topology resulting from the ML analysis of 67 SSU rDNA sequences of cypriniform-infecting myxobolids, plus *Myxidium lieberkuehni* (X76638) and *Zschokkella auratis* (KC849425) as the outgroup. Numbers at the nodes are ML bootstrap values/BI posterior probabilities/MP bootstrap values; asterisks represent full support in all three methodologies; dashes represent a different branching of the BI/MP tree or a bootstrap support value under 50. Dark grey boxes evidence sequences belonging to species that have been reported from the Iberian Peninsula, including the five new sequences presented in this study; the family or sub-family of the vertebrate host is indicated using vertical lines.

Maximum likelihood, BI and MP analyses generated highly similar topologies, with some entropy in the middle of the tree due to the lower ML bootstrap support values of some branches (Fig. 4). The phylogenetic placement of the five new SSU rDNA sequences in study was consistent between phylograms: *Myxobolus arcasii* n. sp. and *M. duriensis* n. sp. clustered in separate branches, but within subclades comprising myxobolids that infect leuciscid hosts; *Thelohanellus paludicus* n. sp. occupied a basal position to several subclades of myxobolids that infect leuciscid and cyprinid hosts; while both sequences obtained for *M. pseudodispar* clustered among the SSU rDNA sequences of other muscle-dwelling parasites of leuciscids and cyprinids worldwide, more specifically with a conspecific sequence from a leuciscid host in Hungary (KU340979). Overall, species sequenced from hosts belonging to Leuciscidae, Cyprinidae and Labeoninae grouped to form multiple distinct subclades within the tree topology.

## Discussion

### Characterization and identification of the myxobolid species

Currently, it is widely accepted that reliable descriptions of myxosporeans can only be the outcome of the combined analysis of several criteria, i.e. myxospore morphology, host and tissue specificity, and molecular data [5, 14, 21]. This is especially true for distinguishing between myxobolids, as myxospores of congener species share great morphological similarity amongst each other, making it essential for descriptions to include additional characters, namely of a molecular nature. The great majority of myxobolids, however, were described mostly based on myxospore morphology [15–18, 79]. Thus, morphology-based comparisons remain necessary to differente between the many species that are without molecular data. Acknowledging that phylogenetic studies widely show the vertebrate host group as the most relevant evolutionary signal for myxobolids (see [7, 21, 62]), the morphological comparisons performed in this study only took into consideration congeners reported from closely related cypriniform species.

*Myxobolus arcasii* n. sp. and *M. duriensis* n. sp. are described from *Achondrostoma arcasii* and *Pseudochondrostoma duriense*, respectively. In terms of phylogeny, the genera *Achondrostoma* and *Pseudochondrostoma* are closely related to each other, as well as to the genera *Chondrostoma* Agassiz, 1832, *Iberochondrostoma*, *Protochondrostoma* and *Parachondrostoma*, representing six lineages of Leuciscidae that, up until recently, were all comprised within *Chondrostoma* [61]. Considering this, the morphological comparison performed here for *M. arcasii* n. sp. and *M. duriensis* n. sp. took into consideration the ca. 118 *Myxobolus* spp. previously described from leuciscid fishes (see [4, 8, 17, 18, 39, 56, 57]), with emphasis on those reported from hosts belonging to the genera *Achondrostoma*, *Chondrostoma* and *Pseudochondrostoma* (see Table 2). To the best of our knowledge, myxozoan parasites have never been reported from *Iberochondrostoma*, *Protochondrostoma* and *Parachondrostoma*. Of the *ca.* 23 *Myxobolus* spp. previously reported from *Achondrostoma*, *Chondrostoma* and *Pseudochondrostoma*, only a few were originally described from these three genera: *M. gallaicus* from *Pseudochondrostoma polylepis* (Steindachner, 1864); *M. leuciscini* simultaneously from *P. polylepis* and *A. arcasii*, but also chub *Squalius cephalus* (Linnaeus, 1758); and *M. arrabonensis* Cech et al., 2015, *M. chondrostomi* Donec, 1962, *M. paksensis* Cech et al., 2015 and *M. szentendrensis* from the common nase *Chondrostoma nasus* (Linnaeus, 1758). Several others were either originally described from hosts of more distant leuciscid genera [*M. albovae* Krasilnikova in Shulman, 1966; *M. bliccae* Donec and Tozyyakova, 1984; *M. bramae*; *M. donecae* Kashkovski, 1969; *M. lobatus* (Nemeczek, 1911) Landsberg and Lom, 1991; *M. macrocapsularis* Reuss, 1906; *M. muelleri* Bütschli, 1882; and *M. pseudodispar*] (Table 2), or from fish species of other cypriniform families (*M. carassii* Klokacheva, 1914; *M. caudatus* Gogebashvili, 1966; *M. circulus* Akhmerov, 1960; *M. cyprini* Doflein, 1898; *M. ellipsoides* Thélohan, 1892; *M. dispar* Thélohan, 1895; and *M. musculi*) prior to being reported from several other cypriniforms, including the leuciscids *C. nasus* and *P. polylepis* [13, 17, 25, 32, 36, 41, 69]. *Myxobolus impressus* Miroshnichenko, 1980 is also included in this list, having been, simultaneously reported from both the leuciscid *Squalius cephalus* and the cyprinid *Barbus barbus* in Ukraine [46]. Considering that most *Myxobolus* spp. are known to be host specific, i.e. they infect only a single or several closely related fish species [6, 20, 49, 52, 54, 57], it is unlikely that *M. carassii*, *M. caudatus*, *M. circulus*, *M. cyprini*, *M. ellipsoides*, *M. dispar*, and *M. musculi* infect fishes of the family Leuciscidae, for which these species were not included in Table 2. *Myxobolus exiguus* Thélohan, 1895, which was also reported to occur in *Chondrostoma nasus* and other Eurasian cyprinids [69], was not considered for morphological comparison, since a recent study disregarded cypriniforms as *bona fide* hosts for this species, now restricted to Mugiliformes [64]. Overall, species comparisons showed *M. arcasii* n. sp. and *M. duriensis* n. sp. differing significantly from all other species reported from leuciscids, be it either in the morphology of the myxospores or molecular data of the SSU rDNA gene. Even though *M. leuciscini* and *M. arcasii* n. sp. share *A. arcasii* as their original host, and there are few morphometric differences between their myxospores, these species could be readily differentiated based on molecular data. *Myxobolus leuciscini* was originally reported from three distinct hosts, and later sequenced from only *Squalius cephalus* (Molnár et al., 2007). Thus, it can be suggested that, as originally described by González-Lanza and Alvarez-Pellitero (1985), *M. leuciscini* probably comprised several morphologically identical species, including *M. arcasii* n. sp.

*Thelohanellus paludicus* n. sp. is the first myxosporean species described from a fish host of the genus *Cobitis*, as well as the first *Thelohanellus* spp. reported from the Iberian Peninsula. Consequently, morphological comparisons to this species were established in relation to the three congeners reported from fish genera of the subfamily Cobitinae worldwide: *T. acuminatus*, *T. misgurni* and *T. pyriformis* (see Table 3). Of the three latter, only *T. misgurni* was originally described from a host of the subfamily Cobitinae (*M. anguillicaudatus*), while *T. acuminatus* and *T. pyriformis* were originally described from hosts of the subfamilies Cyprininae and Tincinae, respectively. Again, it seems unlikely that both these species can infect members of the subfamily Cobitinae. In fact, the high morphological variability found among the various reports of *T. pyriformis* from different tissues and organs of several cyprinids across Europe suggests that it is a possible species complex, despite its status as type species of the genus *Thelohanellus*. As *T. acuminatus*, *T. misgurni* and *T. pyriformis* have not been sequenced from any reported host, molecular comparison to *T. paludicus* n. sp. is impossible; nonetheless, the latter was shown to differ significantly from these three species in both biological and morphological traits.

The study performed here further provides the first combined morphological and molecular report of *M. pseudodispar* from the spleen of *A. arcasii*, and from the muscle, spleen, kidneys and digestive tract of *P. duriense*. Identification of the parasite was based on both the morphological features of the myxospores and molecular data of the SSU rDNA gene. Despite our study showing some genetic diversity between the sequences obtained here from *A. arcasii*, *P. duriense* and those currently available in GenBank for this species, these values were consistent with previous molecular studies that addressed the high intraspecific variability found among different isolates of *M. pseudodispar*. This myxobolid was recently hypothesized to constitute a cryptic species complex, with genetic diversity explained by host-shift followed by ongoing processes of speciation in secondary hosts, and recombination of different lineages in oligochaete hosts (see [24]).

### Phylogenetic analyses

The phylogenetic analyses performed in this study revealed the three new species described here clustering among other cypriniform-infecting myxobolids. This agrees with previous studies that show the vertebrate host group as a relevant evolutionary signal for myxobolids [7, 21, 62]. *Myxobolus arcasii* n. sp. and *M. duriensis* n. sp. specifically cluster among other species that infect leuciscids, but within separate clades, suggesting that myxobolids entered Leuciscidae as hosts multiple times during their evolution. In turn, *Thelohanellus paludicus* n. sp. stands positioned alone at the basis of the leuciscid-infecting clade that contains *M. duriensis* n. sp. This positioning reflects the absence of molecular data belonging to more closely related congeners, as *T. paludicus* n. sp. constitutes the first myxobolid molecularly reported from cypriniforms of the family Cobitidae. Lastly, the SSU rDNA sequences of *M. pseudodispar*, obtained from infections in *A. arcasii* and *P. duriense*, cluster together with their conspecific sequences from Hungarian isolates (here represented by a single SSU rDNA sequence, KU340979 in Fig. 4). Overall, our phylogenetic analyses show that species infecting the same host family cluster together, but not necessarily within the same lineage. Thus, it can be suggested that myxobolids entered different cypriniform families (e.g., Cyprinidae and Leuciscidae) multiple times during their evolution.

Tissue tropism-related phylogenetic clustering has also commonly been reported for myxobolids, as well as myxosporeans in general (see [53] and references therein). Accordingly, the phylogenetic analyses performed during this study placed *M. duriensis* n. sp. among other gill-infecting myxobolids, as well as *M. pseudodispar* among other muscle-dwelling parasites. Nonetheless, *M. arcasii* n. sp. was also shown clustering among several gill-infecting myxobolids, despite its development taking place in the hematopoietic tissue of the kidneys, and in the undifferentiated tissue of the gonads. It may be that *M. arcasii* n. sp. and these gill-infecting species are evolutionarily related by having tropism to tissues with high rates of regeneration and demanding nutrient intake; however, it is also likely that the positioning of *M. arcasii* n. sp. reflects the influence of one or more stronger evolutionary signals. Overall, tissue tropism is most likely an important fine-scale evolutionary signal for myxobolids. However, the lack of information regarding the specific tissue of development of most species hampers recognition of its real influence on the evolution of these parasites. Furthermore, it cannot be disregarded that the evolutionary signals currently accepted as having played a preponderant role in the evolution of myxobolids are based on “mixed signals” of invertebrate and vertebrate co-phylogeny [30]. Thus, the discernment of phylogenetic patterns related to the invertebrate host may reveal other evolutionary signals that were decisive in the evolution of these parasites.

### Myxobolid biodiversity in endemic Iberian cypriniforms

A review of the available literature found 12 myxosporean species that have been reported from cypriniform hosts in the Iberian Peninsula. Among these, 11 belonged to the genus *Myxobolus*: *M. branchilateralis*, originally described from the gills of the common barbel *Barbus barbus* (Linnaeus, 1758) in Hungary, and simultaneously reported from *Luciobarbus bocagei* (Steindachner, 1864) in Portugal; *M. branchialis*, originally described from the gills of *B. barbus* in Ukraine, and later reported from the same fish host in Hungary and *L. bocagei* in Portugal; *M. cutanei* from the scales of *L. bocagei* in both Spain and Portugal; *M. gallaicus* and *M. leuciscini* from the gills of the Iberian nase *P. polylepis* in Spain; *M. muelleri*, a species complex that gained identity through its morphological and molecular re-description from the gills of chub *Squalius cephalus*, but possibly occurs in several other reported cypriniform hosts, including *A. arcasii* and *P. polylepis* in Spain; *M. impressus*, originally described from the fins and gills of *B. barbus* and *S. cephalus* in Ukraine, and later reported from the gills of *P. polylepis* in Spain; *M. musculi*, a muscle parasite of *B. barbus* in Hungary and *L. bocagei* in Portugal; *M. pfeifferi*, originally described from the connective tissue of several organs of *B. barbus*, and later reported from *L. bocagei* in Portugal; *M. pseudodispar* from the muscle of roach *Rutilus rutilus* and several other cyprinids in Europe, including *P. polylepis* and *S. cephalus* in Portugal; and *M. tauricus*, originally described from the gills, fins and muscles of the Crimean barbel *Barbus tauricus* Kessler, 1877 in Ukraine, and later reported from the pin bones and fins of *B. barbus* in Hungary and *L. bocagei* in Portugal (see [1, 2, 10, 17, 18, 25, 32, 55, 57, 67, 74]). The remaining non-myxobolid species, *Myxidium rhodei* Léger, 1905, was reported from the kidneys of *P. polylepis* and *S. cephalus* in Spain [1].

Cypriniforms are mostly restricted to freshwater and can naturally expand their distribution only through the direct connection of habitats [66, 72]. The translocation of known host species into new geographic areas therefore plays an important role in the dissemination and establishment of cypriniform-infecting parasites. The common carp *Cyprinus carpio*, for instance, is largely recognized as being responsible for numerous co-introductions and co-invasions of different parasitic groups worldwide, including myxozoans (e.g. [50, 60, 68, 71, 76]). Reported cases of co-introduction with *C. carpio* include the myxobolid species *Thelohanellus hovorkai* Akhmerov, 1960 and *T. nikolskii* Akhmerov, 1955, both originally from the Amur basin and later introduced to Hungary [48, 50]. In the same manner, the human-mediated translocation of the silver carp *Hypophthalmichthys molitrix* (Valenciennes, 1844) and bighead carp *Hypophthalmichthys nobilis* (Richardson, 1845) to Hungary from Eastern Asia in the early 1960s, was reported to have led to the co-introduction of *Myxobolus pavlovskii* (Akhmerov, 1954) Landsberg and Lom, 1991 [17, 45, 47]. In this study, the occurrence of *M. pseudodispar* in endemic Iberian cypriniforms provides evidence for another case of parasite/host co-introduction.

*Myxobolus pseudodispar* is one of the most common muscle-dwelling parasites of leuciscids in Europe, having originally been described from roach *Rutilus rutilus*, and since then reported from several other species, including rudd *Scardinius erythrophthalmus*, freshwater bream *Abramis brama*, common bleak *Alburnus alburnus*, and white bream *Blicca bjoerkna* [24, 54]. All these fish species were introduced into the Iberian Peninsula during the 20th century [28, 35], where *M. pseudodispar* was reported to occur in several organs of the endemic species *A. arcasii* and *P. polylepis*, and the native species *S. cephalus* [10, 25]. In this study, *M. pseudodispar* is further reported from the endemic species *P. duriense*, therefore adding a new host record to this parasite in the Iberian Peninsula. Despite strict host specificity being frequently reported among myxobolids [6, 20, 49, 52, 53, 55, 57], several species of *Myxobolus*, including *M. pseudodispar*, *M. bliccae* and *M. macrocapsularis*, have been reported to have host-shifted between members of the cypriniform family Leuciscidae [24, 52, 57]. Considering this, it is possible that *M. pseudodispar* was co-introduced into the Iberian Peninsula with different leuciscid hosts on multiple occasions. Nonetheless, further research on endemic and exotic species is necessary in order to understand the origin and dispersion of *M. pseudodispar* in the Iberian Peninsula, a task that will certainly prove difficult given the continuous introduction of potential cypriniform hosts into this geographical region during the past century [28, 35]. The acquisition of this knowledge would, moreover, be relevant for understanding how the cryptic speciation of *M. pseudodispar* relates to its adaptation to distinct micro- and macroenvironments.

Similarly to *M. pseudodispar*, several other species of the above-mentioned *Myxobolus* spp. reported from Iberian endemic cypriniforms were originally described from central and eastern European species; such are the cases of *M. branchialis*, *M. branchilateralis*, *M. musculi*, *M. pfeifferi* and *M. tauricus*, originally described from either *B. barbus* or *B. tauricus* and, since then, reported from the Iberian endemic species *Luciobarbus bocagei*. Nonetheless, the occurrence of most of these species in *L. bocagei* has not been corroborated by molecular data. Reports of *M. branchialis* and *M. branchilateralis* from *L. bocagei* lack molecular data for comparison to the SSU rDNA sequences available from Hungarian isolates in *B. barbus*. In contrast, *M. pfeifferi* was sequenced from *L. bocagei* but never from its original host *B. barbus*. Molnár et al. [55] provided molecular data for *M. tauricus* from both Hungarian isolates in *B. barbus* and Portuguese isolates in *L. bocagei*; however, given the significant genetic variability found between geographical isolates, the authors themselves suggested that they were possibly dealing with two distinct species. Thus, *Myxobolus musculi* constitutes the only species whose identification from infections in *L. bocagei* was molecularly substantiated by means of comparison to the data available in GenBank from isolates belonging to other geographical areas. Nonetheless, the high genetic variability found between Hungarian isolates of *M. musculi* in *B. barbus* and Portuguese isolates in *L. bocagei*, as well as between isolates belonging to the same geographic location, suggests that future cross-infection experiments are necessary to confirm putative host-shift of this species, especially since there is no record of the introduction of *B. barbus* into the Iberian Peninsula.

Overall, myxosporean richness in the Iberian Peninsula appears to be underestimated, with the present study describing three new and one known species from endemic cypriniform hosts. As such, it is suggested that myxozoan research in this geographic region be expanded to target a broader array of endemic, native and non-native species. New myxozoan surveys of *L. bocagei*, specifically, are necessary in order to ascertain the identity of the myxobolids previously reported from this host, as it is plausible that some will constitute new species records.
